# Defining the Human-Biota Thresholds of Toxicological Concern for Organic Chemicals in Freshwater: The Proposed Strategy of the LIFE VERMEER Project Using VEGA Tools

**DOI:** 10.3390/molecules26071928

**Published:** 2021-03-30

**Authors:** Diego Baderna, Roberta Faoro, Gianluca Selvestrel, Adrien Troise, Davide Luciani, Sandrine Andres, Emilio Benfenati

**Affiliations:** 1 Laboratory of Environmental Chemistry and Toxicology, Istituto di Ricerche Farmacologiche Mario Negri IRCCS, Via Mario Negri 2, 20156 Milano, Italy; roberta.faoro@studenti.unimi.it (R.F.); gianluca.selvestrel@marionegri.it (G.S.); davide.luciani@marionegri.it (D.L.); 2 INERIS Institut National de l’Environnement Industriel et des Risques, Rue Jacques Taffanel, 60550 Verneuil-en-Halatt, France; Adrien.TROISE@ineris.fr (A.T.); Sandrine.ANDRES@ineris.fr (S.A.)

**Keywords:** threshold of toxicological concern, eco-TTC, human-biota TCC, chemical risk assessment, screening levels, toxicology

## Abstract

Several tons of chemicals are released every year into the environment and it is essential to assess the risk of adverse effects on human health and ecosystems. Risk assessment is expensive and time-consuming and only partial information is available for many compounds. A consolidated approach to overcome this limitation is the Threshold of Toxicological Concern (TTC) for assessment of the potential health impact and, more recently, eco-TTCs for the ecological aspect. The aim is to allow a safe assessment of substances with poor toxicological characterization. Only limited attempts have been made to integrate the human and ecological risk assessment procedures in a “One Health” perspective. We are proposing a strategy to define the Human-Biota TTCs (HB-TTCs) as concentrations of organic chemicals in freshwater preserving both humans and ecological receptors at the same time. Two sets of thresholds were derived: general HB-TTCs as preliminary screening levels for compounds with no eco- and toxicological information, and compound-specific HB-TTCs for chemicals with known hazard assessment, in terms of Predicted No effect Concentration (PNEC) values for freshwater ecosystems and acceptable doses for human health. The proposed strategy is based on freely available public data and tools to characterize and group chemicals according to their toxicological profiles. Five generic HB-TTCs were defined, based on the ecotoxicological profiles reflected by the Verhaar classes, and compound-specific thresholds for more than 400 organic chemicals with complete eco- and toxicological profiles. To complete the strategy, the use of in silico models is proposed to predict the required toxicological properties and suitable models already available on the VEGAHUB platform are listed.

## 1. Introduction

Toxicological profiling of substances released into the environment is essential to assess the risk of adverse effects on human health, following different routes of exposure. The process is expensive and time-consuming and, today, the information available for so many compounds is still partial. The situation is even more complicated if we consider the adverse effects on ecological receptors and the environment in all its complexity.

To facilitate the hazard and risk assessment of chemicals, whether recently developed or already available on the market, the concept of Threshold of Toxicological Concern (TTC or hTTC) was introduced in the 90s [1,2]. TTC is a generic human exposure threshold below which there would be no appreciable health effects and it is generally used when there are no chemical-specific toxicity data [3,4,5]. Nowadays, hTTC is a consolidated approach in risk assessment to estimate the dangers of chemicals from different routes and sources of exposure such as diet and inhalation [6,7,8,9,10,11], or for specific groups of contaminants [12,13,14,15,16,17,18]. Similar to what was proposed for human health, a threshold approach called Ecotoxicological Threshold of Toxicological Concern (eco-TTC) has also been recently proposed for the environmental risk assessment [19,20,21]. These thresholds are based on the Predicted No effect Concentration (PNEC) data retrieved for chemicals sharing the same mode of toxicological action and are set equal to the most toxic values among each group to derive a safe concentration suitable for the entire class [19,20,21].

Traditionally human and ecological risk assessments are conducted in parallel but separately [22,23]. Only a few attempts have been made to integrate the two procedures, particularly for environmental contaminants, such as the ERICA index [24].

The LIFE VERMEER project (LIFE16 ENV/IT/000167—www.life-vermeer.eu, (accessed on 1 March 2021)) wants to offer a comprehensive platform for the integrated risk assessment of chemicals, focusing on the identification and substitution of risky substances. The aim is achieved by combining existing software for exposure and hazard assessment and the definition of an integrated strategy for human and environmental risk assessment of chemicals used in some of the most important industrial sectors with large and increasing environmental impacts such as cosmetics and personal care products, food contact materials, solvents, dispersants, and biocides. The project pays particular attention to the definition of Human-Biota TTCs (HB-TTCs) as the alarm levels to be used in the first screening of hazards induced by chemicals released into the environment.

The aim of this study was to develop a strategy for the definition of generic and compound-specific HB-TTCs for the aquatic environment as safe freshwater concentrations for human and ecological receptors at the same time, using public data and the in silico tools implemented in the VEGAHUB platform (www.vegahub.eu (accessed on 1 March 2021)).

To do this, we linked the xenobiotics’ concentration in freshwater to ecological and toxicological effects using eco- and toxicological-information and possible exposure scenario. Differently from for aquatic organisms, man is not directly exposed to the pollutants present in fresh waters and it is therefore necessary to define an exposure scenario that describes a realistic route of exposure for man which, in our case, consists in the water use for drinking purpose.

Eco- and toxicological-features of pollutants were described by specific profilers to define preliminary screening thresholds for compounds with no eco- and toxicological-information available while detailed, compound-specific parameters were used for those chemicals with known hazard assessments in terms of no effect values for freshwater ecosystems and acceptable doses for human health.

## 2. Results

Here we describe the data distribution in the eco- and toxicological-classes, the proposed eco-TTCs and the general HB-TTCs.

### 2.1. Preliminary Integrated Profile for Human and Ecological Risk Assessment

**Figure 1:**
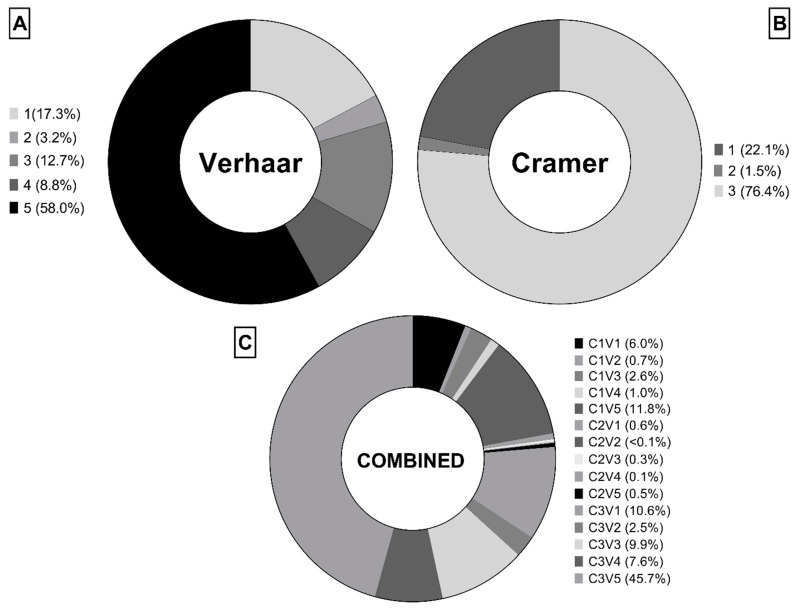
Distribution of chemicals (*n* = 3728) in the curated EnviroTox: (**A**) Verhaar classes, (**B**) Cramer classes, (**C**) combined classification (C = Cramer, V = Verhaar).

The use of profilers allowed the grouping of the substances according to their preliminary eco- and toxicological-profile (Figure 1). The classification of chemicals is the first step to derive screening values for chemicals of interested without performing any additional toxicity testing. We retrieved structural information and PNEC data for 3728 organic chemicals from the freely available EnviroTox platform of the Health and Environmental Sciences Institute (HESI) [20,25,26]. According to the Verhaar classification [27,28], most of the chemicals in this dataset cannot be classified (class 5) while narcosis (class 1) and unspecific reactivity (class 3) are the two most common modes of action (Figure 1A). Focusing on human toxicology, more than two thirds of chemicals fell into Cramer class III (Figure 1B) [29]. The combined classification accounting of Verhaar and Cramer classes (Figure 1C) gave 15 possible combinations: most of the chemicals were classified as “Cramer class 3 and Verhaar class 5” (C3V5, 45.7%), followed by “Cramer class 1 and Verhaar class 5” (C1V5, 11.8%) and “Cramer class 3 and Verhaar class 1” (C3V1, 10.6%).

### 2.2. Proposed Eco-TTCs and Quality Standards from hTTCs.

Profiling the dataset for Verhaar classification was necessary to group chemicals according to their mode of action on aquatic organisms. This was fundamental for the derivation of eco-TTCs. We selected the Verhaar classification because it is a consolidated approach and because it is possible to classify the chemicals of concern automatically with public available profilers such as Toxtree or VEGAHUB implementing the rationale behind the classification.

The eco-TTC of each subset based on the Cramer classification was calculated as the fifth percentile of the log10-transformed PNEC distribution. Empirical, normal fitted and logistic fitted distributions were calculated (Table 1).

Safer concentrations for humans were defined according to the Water Framework Directive and selecting the water abstracted for drinking purpose as exposure route for humans. In this way, the water Quality Standards (QS) for the protection of human health have been defined considering both the toxicology of the contaminants, expressed by Cramer classification, and a possible exposure scenario. The Cramer decision-tree is based on relationships between specific structures and metabolic pathways leading to toxicity. The classification includes three classes from low potential toxicity (class I) to significant toxicity (class III) and a TTC value is associated to each class. More details are listed in the Section 4. The (QS) derived from the hTTCs are shown in Table 2. For each class, two values are proposed because of different body weight in the Water Framework Directive (WFD, 70 kg) [30,31] and the TTC approach (60 kg).

### 2.3. General Human-Biota TTCs (HB-TTCs)

In the proposed approach, the lowest value between the proposed eco-TTC and hTTC-derived QS for each chemical was selected as Human-Biota TTCs.

The combination of the two classifications resulted in 15 possible combinations as a result for 3 human classes and 5 ecological profiles (see Supplementary Material SM2). In our experimental conditions, the eco-TTC was always lower than the hTTC derived concentration for each combination, so the generic HB-TTC was equal to the five above-mentioned eco-TTC (Table 3).

As an example, the HB-TTC 5.496 ng/L is valid for each chemical profiled as Verhaar class 1, independently from the results of Cramer profiling.

### 2.4. HB-TTCs Specific for Each Chemical

The generic HB-TTCs listed in Table 3 can be applied to chemicals with no eco- and toxicological data as benchmarks for preliminary screening. Compound-specific HB-TTC can be obtained when experimental data relating to the toxicological and ecotoxicological potential are available or, as we will see later, when predictions are available for the properties of interest.

**Figure 2:**
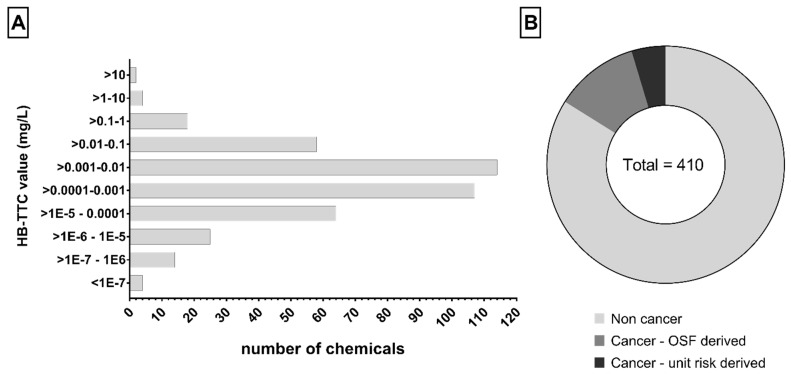
Compound-specific HB-TTCs for 410 chemicals: (**A**) distribution of values, (**B**) toxicological features. OSF = Oral Slope Factor.

We derived 410 HB-TTCs combining data from the EnviroTox database, the No Observed Adverse Effect Level (NOAEL) dataset collected from the literature, and data for carcinogenic compounds (see Supplementary Material SM3). Values range from 2.996 × 10^−8^ to 20.955 mg/L and the distribution in fixed intervals is shown in Figure 2A. Focusing on the types of chemical, most of the compounds are non-carcinogenic (344, ~84%) (Figure 2B).

## 3. Discussion

The number and volume of chemicals released into the environment from natural and anthropogenic activities make the assessment of their potential adverse effects on human health and environment more essential day by day. Information for this assessment is still lacking for most of the chemicals and getting this data from testing takes time and money. In addition, under the Water Framework Directive, there is no regulatory leverage to require experimental data for either prioritization of substances of concern for environmental quality standards. The Thresholds of Toxicological Concern (hTTC), a concept introduced in the 1990s [1,2,8], are used in case of missing data as a preliminary human exposure threshold values considered safe below which no appreciable adverse effects on health are expected [3,4]. The TTC approach was recently proposed also for environmental risk assessment, applying the so called Ecotoxicological Threshold of Toxicological Concern (eco-TTC) but studies on this topic are still limited [19,20,21]. Both these approaches are based on chemicals with well-known eco- and toxicological profiles grouped according to their common mechanisms of action and toxicity levels. Though the approach is consolidated for risk assessment of chemicals, the use of TTC is still questioned because these general safe concentrations are considered too conservative. Moreover, considering that risk assessment is conducted separately for humans and the environment, the procedure could indicate different safer concentrations.

In this study, we propose an approach for the definition of human-biota-TTCs (HB-TTCs) for the aquatic environment. HB-TTCs can be considered safe freshwater concentrations for human and ecological receptors. Two series of HB-TTCs were defined: the first set are generic concentrations for chemicals grouped according to their mode of action or toxicity level, while the concentrations in the second group are compound-specific thresholds based on their specific profiles in terms of safe concentrations or doses (such as NOAEL, PNEC, drinking water concentration corresponding to a defined cancer risk). To define the first set of thresholds, the Cramer and Verhaar profilers, respectively for the toxicological and ecotoxicological classification of substances, play a fundamental role in the adopted approach while the second type is based only on the specific toxicity of each substance.

### 3.1. The Proposed Eco-TTCs

An essential step in the proposed strategy for generic HB-TTCs is to define eco-TTCs as generic safe concentrations for freshwater organisms, according to their common mode of action.

To do this, we profiled the organic chemicals in the HESI EnviroTox database, which includes PNEC values for freshwater organisms. Chemicals were analyzed with the VEGA implementation of the Verhaar scheme as it appears in the Toxtree software, and grouped in 5 sets according to the defined classes. The Verhaar classification is a consolidated approach used worldwide to classify substances according to their ecotoxicological mode of action [27,28]. Then the fifth percentile of the log10-transformed PNEC distribution of each set was calculated and selected as eco-TTCs for the class, similarly to what is done for the definition of human TTC [1,2,3,4,5].

Table 4 compares the eco-TTCs found in our experimental conditions with those obtained by Kienzler and collaborators on the EnviroTox database [21]. The eco-TTCs in this study for Verhaar classes differs from those previously reported: in particular, values for classes 1, 2 and 3 are about one order of magnitude lower while for class 4 the value we propose corresponds to about 60% of that reported in the literature. Finally, values for compounds in class 5 are comparable. In our opinion the differences found can be traced to 3 main factors: (1) our study focuses only on organic substances while the other study also includes inorganic compounds, (2) the different number of compounds considered and (3) the different profiling of substances, which results in a different distribution of the compounds in the 5 classes.

This last point, which we believe has the greatest impact, is because the mode of action proposed by Kienzler and collaborators is a consensus of several profilers [21] while our approach used a single profiler (though this is one of the profilers used in the consensus assessment).

### 3.2. Generic Human-Biota TTCs

Generic freshwater HB-TTCs could be employed in the risk assessment of chemicals without chemical-specific eco- and toxicological data as for the TTCs [3,4,5]. In our framework, in facts, they indicate a concentration of the chemical in freshwater below which no appreciable adverse effects occur on human or ecological receptors. The safety of these concentrations is based on the combination of eco-TTC for freshwater organisms and the drinking quality standards based on human risk assessment from the more consolidated human TTCs, according to the approach described by the Water Framework Directive guidance document [31]. The two concentrations are then compared, and the lowest value is defined as the HB-TTC for the chemicals sharing the same eco- and toxicological profiles. In this way, it is conceivable that contamination values below those proposed are not capable of inducing adverse effects on the health of aquatic organisms and of humans who consume water captured by the water body for drinking purpose. This is justified by the fact that the proposed values derive from no-effect concentrations for the aquatic environment as expressed by PNEC and no-effect doses for humans as established by the TTC concept. Fifteen possible integrated profile combinations result from the merge of the 5 Verhaar classes and the 3 Cramer classes but, in our experimental conditions, only 5 HB-TTC values are defined because the eco-TTC we propose are always lower than the QS values derived from the human TTC as defined by Cramer [29]. The ecotoxicological classification therefore guides the process of defining generic HB-TTCs and, in the absence of a complete eco- and toxicological profile, the safe concentrations for aquatic organisms are also abundantly safe for potential adverse effects induced in humans following consumption of water abstracted for drinking purpose. This is because the concentrations defined by the ecological branch of the proposed strategy are always the lowest concentrations, regardless of the association with the concentrations relating to Cramer classes 1, 2 or 3. This conclusion is also valid if the eco-TTCs proposed by the work of Kienzler and collaborators are considered [21].

In the proposed work, we focused on the water abstracted for drinking purpose as exposure route for humans, according to the WFD guidance. Other routes of exposure to contaminants present in water are possible: the same WFD, for example, also introduces the consumption of fish products raised or fished in freshwaters. This approach was previously used for the ecological and human exposure assessment to PBDEs in Adige River using the MERLIN-Expo platform to model the ecological scenarios for invertebrates and fishes and the human scenario based on ingested fish products [32]. In addition, researchers included also a physiologically based pharmacokinetic (PBPK) model to estimate the fate of the ingested contaminants in human body [32]. The use of these models could be used in the future to refine the proposed HB-TTC values with deeper focus on compost-specific values.

### 3.3. Compound-Specific Human-Biota TTCs

The proposed strategy allowed us to derive specific HB-TTCs for 410 chemicals, including carcinogens and non-carcinogens. The proposed values range from 3 × 10^−8^ to 21 mg/L (Figure 2) and most fall in the range 0.1–10 µg/L (221, ~54%).

While generic HB-TTCs can be used as screening level in a preliminary risk assessment, compound-specific HB-TTCs are proposed for detailed risk assessment in a One Health perspective. In our proposed strategy these safer concentrations result from the integration of eco- and toxicological data on the compound of interest, such as PNEC for freshwater organisms and the drinking quality standards derived from NOAEL, oral reference dose, oral slope factors and drinking water concentration corresponding to specific cancer risk level. All these parameters are currently used in chemical risk assessment. The specific HB-TTCs were set at the lowest concentration between the PNEC and the quality standards: this is an important difference from what was done for generic thresholds, though it shares the basic conceptual strategy.

The adoption of specific parameters for substances in place of information from grouping and macrofamilies gives less-conservative thresholds, tailored to the specific toxicological profiles of the chemicals investigated. Focusing on the list we propose, this assumption is valid for 378 (more than 92%) of the 410 specific HB-TTCs (see Supplementary Material SM3).

As in the case of general thresholds, the concentration from the ecotoxicological branch was selected, in most cases, as the HB-TTC value as it was lower than that obtained from the human toxicology. However, it is important to note that in the compounds identified as carcinogens, HB-TTC comes from the toxicological front: out of 66 compounds, 53 have thresholds from human toxicology lower than those established by the ecotoxicology branch of the strategy.

### 3.4. Use of VEGA in Silico Models for the Predictions of Eco- and Toxicological Properties of Substances with Partial Toxicological Information

In the present work, we propose two strategies for the definition of HB-TTCs for organic chemicals, with a general or compound specific approach. For substances other than those listed in Supplementary Material SM3, it is always possible to derive the general HB-TTCs by profiling with profilers already available and widely used, while the compound-specific approach requires more data, not always available. When data is lacking, one can use in silico models to predict the eco- and toxicological properties of interest [33,34,35]. These data will then be integrated with any experimental data available to complete the toxicological assessment. The currently available in silico ecotoxicological models are not able to provide predictions for the PNEC directly but these tools predict single endpoint for each of the three trophic levels considered. Therefore, it is necessary to derive the PNEC from these predictions by applying appropriate safety factors, taking account of the reliability of the prediction. A non-exhaustive list of usable in silico models is shown in Table 5.

Focusing on VEGAHUB, several models are already freely available for the properties listed in our strategy and can be used for organic chemicals only.

The NOAEL model is a QSAR model based on data from repeated dose 90-day oral toxicity studies in rodents and the graph of atomic orbitals by the Monte Carlo method, using CORAL software [61].

A combined approach for carcinogenicity based on oral slope factors has been recently uploaded on the platform. It includes a classification model to distinguish between carcinogens and non-carcinogens and a regression model for the quantitative prediction of the oral slope factor [62].

The algae toxicity models are a Tree Ensemble Random Forest built respectively with 72 h EC50 data based on growth rate and 72 h NOEC values from the Japanese Ministry of Environment, respectively for the acute and chronic models.

The two models for acute toxicity with *Daphnia magna* are both based on 48 h EC50 data from studies following the OECD 202 guideline: the DEMETRA model is a hybrid model based on multiple linear regression and the IRFMN model is a Tree Ensemble Random Forest.

The Daphnia Chronic toxicity model is a Tree Ensemble Random Forest based on experimental 21 d NOEC data retrieved from the Japanese Ministry of Environment and selected according to the OECD TG 211 guideline.

Several models for acute fish toxicity are implemented in VEGAHUB. For the purpose of this work, we suggest the following models:

- The NIC model, a Counter Propagation Artificial Neural Network (CP-ANN) [63] with 96 h LC50 values for *Oncorhynchus mykiss, Oryzias latipes, Pimephales promelas* and *Poecilia reticulata*;
- The IRFMN model, a Tree Ensemble Random Forest with 96 h LC50 data from studies with *Oryzias latipes* following OECD 203;
- The Fathead minnow model, a k nearest neighbor (kNN) model with 96 h LC50 data with *Pimephales promelas*.

Finally, the fish chronic toxicity model is a Tree Ensemble Random Forest built with data from the early-life stage toxicity test (OECD TG 210) and juvenile growth test (OECD TG 215) collected by the Japanese Ministry of Environment.

It is important to note that all the models implemented in VEGAHUB gives an overall assessment of the reliability of the provided predictions based on several parameters relating to the results on similar chemicals in the training and test sets of the model [63]. This information includes the correctness of the prediction on similar compounds, the consistency between the predicted value for the target compound and the experimental values of the similar compounds, the range of the descriptors, and the presence of unusual fragments, using atom centered fragments [64]. These elements are used by VEGA to identify possible reasons of concern, and thus to characterize the uncertainty of the results. The predicted values have a lower level of reliability compared to the experimental values, which should be preferred whenever available. If the substance of concern has a predicted value of low reliability, the user should have a look at the experimental values of the similar compounds, which are provided by VEGA.

Further details about the above models are reported in Supplementary Materials SM4 and for a general discussion of the VEGA models for ecotoxicological properties see [48].

## 4. Materials and Methods

The first part of this section gives the approach for the definition of generic HB-TTCs and the strategy applied for compound-specific HB-TTCs is described in the second part.

**Figure 3:**
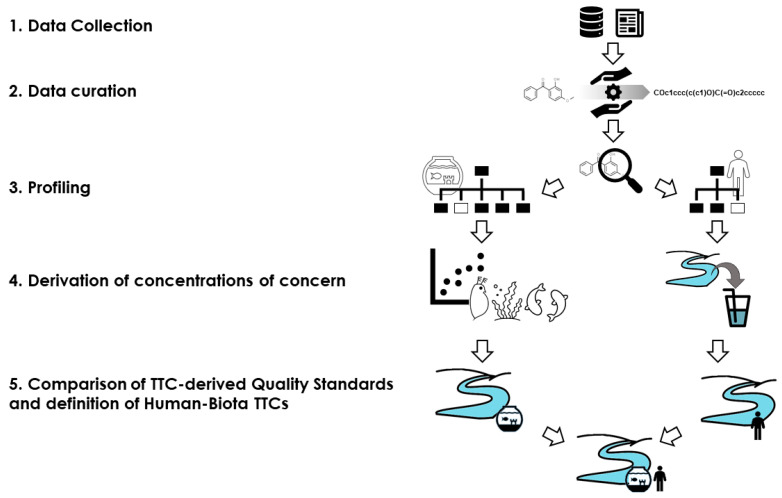
Scheme of the strategy to obtain general Human-Biota TTCs.

The proposed strategy for general HB-TTCs is summarized in Figure 3.

### 4.1. Data Collection

Ecotoxicological data for freshwater species were collected from the EnviroTox database by the Health and Environmental Sciences Institute (HESI) [20,26]. The collection includes experimental data from acute and chronic aquatic toxicity tests on algae, invertebrates, and fish, associated to Canonical SMILES for each chemical. Predicted No-Effect Concentration (PNEC) values were extracted automatically, applying the PNEC Calculator tool included in the platform on the entire downloadable database: briefly, for each chemical, the tool calculates the geometric mean of multiple data for the same endpoints (the combination of acute or chronic and algae, invertebrates, and fish) and then the PNEC is calculated considering the European chemical assessment conditions implemented in EnviroTox [26]. The Assessment Factors (AFs) used are reported in Table 6.

No Observed Adverse Effect Level (NOAEL) data from repeated dose 90-day oral toxicity studies in rats were collected from several public repositories, in line with their adherence to the OECD 408 guideline [65]. The sources of data include the Munro database and the Hazard Evaluation Support System (HESS) database, both available from the OECD QSAR Toolbox [66,67], the Integrated Risk Information System (IRIS) by the U.S. Environmental Protection Agency [68], the COSMOS database [17], the U.S. EPA’s ToxRefDB [69] and the EFSA’s OpenFoodTox Database [70,71]. These data were used only to obtain quality standards from human TTC in the strategy for compound-specific Human-Biota TTC.

### 4.2. Data Curatiom and Profiling

Inorganic and organometallic compounds, polymers, isomeric mixtures, and data relating to mixtures of chemicals were removed from the ecotoxicological and NOAEL datasets, as previously reported [72]. Where necessary, structures were desalted manually using the Marvin suite by Chemaxon [73].

From the data curation, a final dataset with ecotoxicological data relating to 3728 chemicals (curated EnviroTox DB) and a NOAEL dataset of 746 compounds were obtained.

Chemicals in the curated EnviroTox DB were first analyzed with the Cramer rules module of Toxtree [3,29] as recently implemented in the VEGAHUB platform (www.vegahub.eu (accessed on 25 February 2021)). This decision tree estimates the human TTC (hTTC) for a substance based on its chemical structure. As a result, the substance is classified in one of the three Cramer classes where class I is for chemicals with low oral toxicity while class III includes chemicals with the most severe toxic hazard (Table 7).

The curated EnviroTox DB were then profiled with the VEGA implementation of the Verhaar scheme in Toxtree software (version 3.2, Joint Research Center, European Commission, Ispra, Italy) [27,28]. The profiler classifies substances in 5 classes according to their Mechanism of Action (MoA) of substances for fish acute toxicity (Table 8).

### 4.3. Derivation of Concentrations of Concern

This section describes the data processing to obtain the hTTC-derived water quality standards and the eco-TTCs.

#### 4.3.1. Eco-TTC Derivation

To compute the eco-TTC, the fifth percentile of the ecotoxicological values was calculated, similarly to what is done to give the human TTC [1,2,20,74]. Five subsets of PNECs were created according to the Verhaar profiling. For each subset, the fifth percentile of the log10-transformed PNEC distribution was calculated. Three approaches were applied: calculation on the empirical distribution, by fitting normal distribution and by fitting logistic distribution. All this was done with R [75] and the script is reported in Supplementary Materials (SM1). As the final step, the fifth percentile from these distributions were back-transformed to the original concentration.

#### 4.3.2. Reverse Approach to Obtain Quality Standards for Water Abstracted for Drinking Purpose

The European Water Framework Directive (WFD) [30] requires quality standards (QS) to protect humans against substances in water bodies used for the abstraction of drinking water. For this scope, the Technical Guidance no 27 For Deriving Environmental Quality Standards (QS) [31] provides the following equation to derive quality standards for drinking-water abstraction (Equation (1)):

QS = (0.1 × TL × bw)/wu
(1)

where TL is the human toxicological standard, set equal to each human TTC values as defined by Cramer, (bw) is the Body Weight (70 kg) and (wu) is the daily Water Uptake (2 L).

Our proposed approach uses the equation 1 to work out the water concentration from the hTTC values or from other toxicological values, which are doses in humans, not environmental concentrations.

### 4.4. Comparison of TTC-Derived Quality Standards and Definition of Human-Biota TTCs

As stated before, the eco-TTC and the hTTC-derived quality standards are both water concentrations to protect respectively freshwater ecosystems and human health. To define the Human-Biota TTC as a toxicant concentration in freshwater protecting at the same time both humans and ecosystems, we selected the lowest values between the proposed eco-TTC and hTTC-derived QS.

### 4.5. Strategy for the Definition of Compound-Specific Human-Biota TTC

**Figure 4:**
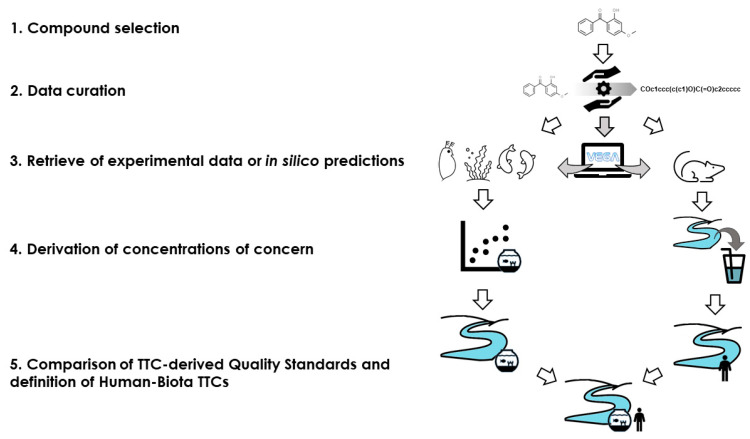
Scheme of the strategy to derive compound-specific Human-Biota TTCs.

In this section, the strategy for obtaining HB-TTC specific for each chemical is described (Figure 4). Unlike what has been described so far, the approach uses specific eco- and toxicological data for each chemical considered, instead of the profilers for the Verhaar and Cramer classes.

As for the general approach, each chemical was characterized by name, CAS number, and SMILES. This facilitates the search for compounds of interest in both datasets and allows the use of in silico models to predict missing data.

The PNEC value from the EnviroTox database was used directly for the assessment of the ecotoxicological properties while a more complex approach was applied to obtain quality standards for human health.

For the identification of carcinogens, chemicals with a defined oral slope factor (SF) in the the IRIS and RAIS lists were considered carcinogenic and those with no SF value were considered non-carcinogenic based on the available data [62].

In the case of noncarcinogenic toxic chemicals, the acceptable daily intake (ADI), the tolerable daily intake (TDI), the oral reference dose (RfD) or the benchmark dose can be used directly as human toxicological standard while the NOAEL values need to be processed as described in Equation (2):

TL = NOAEL/100
(2)

For carcinogens, the QS was set equal to the drinking water concentration corresponding to a cancer risk level of 1 in a million (1 × 10^−6^) additional case as acceptable cancer risk level. This toxicological value was retrieved from the IRIS database or derived from the slope factors from the Risk Assessment Information System (RAIS) [76] with Equation (3):

QS = [(1 × 10^−6^/SF) × bw ]/wu (3)

where 1 × 10^−6^ is the acceptable cancer risk, SF is the oral slope factor, bw is the body weight (70 kg) and wu is the daily water uptake (2 L).

As example, a dataset of 410 chemicals was processed. This dataset included chemicals present in both EnviroTox and NOAEL databases in order to retrieve easily the eco- and toxicological properties of interest.

As for the general approach, the HB-TTC specific for each chemical was defined by comparing the eco-TTC and the hTTC-derived quality standards and selecting the lowest values between them.

## 5. Conclusions

The goal of chemical risk assessment is to have a full understanding of the nature of a potential adverse health or environmental effect induced by a chemical and to provide an estimation of the magnitude and probability of these risks in the target organisms. The eco- and toxicological profile of a substance plays a fundamental role in this procedure but obtaining the necessary data for a correct and detailed hazard assessment of a substance takes time and money. A consolidated approach to overcome this problem is the use of TTCs for the assessment of the potential health impact and, more recently, of eco-TTCs for the ecological aspect. These are considered safe concentrations or doses for substances with poor toxicological characterization and low exposure. Although the One Health approach recognizes a link between humans, organisms and their shared ecosystem, human and ecological risk assessments are traditionally conducted in parallel but separately.

In this paper we proposed a strategy to define the Human-Biota TTCs as concentrations of organic chemicals in freshwater, safe for both humans and ecological receptors. The threshold value is defined by the combination of the eco- and toxicological profile of the substance and the exposure scenario, required to translate a safe dose for humans into a maximum allowable concentration of the substance in the water that does not cause adverse effects in humans who capture that water for drinking purposes.

Two sets of thresholds were provided: general HB-TTCs as preliminary screening levels for compounds with no eco- and toxicological information available, and compound-specific HB-TTCs for those chemicals with known hazard assessment in terms of PNECs for freshwater ecosystems and acceptable doses for human health.

The first set of values is based on the integration of information deriving from the eco- and toxicological classification of the substances of interest through validated and internationally used profilers. No additional (eco)toxicological tests are therefore required, and the assessment derives from pre-existing information on substances that share the same mechanism of action as the test substance. The use of publicly available profilers (e.g., Toxtree and VEGAHUB) allows substances to be classified automatically, reproducibly and independently of the risk assessor. On the other hand, for the definition of the second set of values, information on the specific toxicity of the test substance such as NOAEL or PNEC is required. These values can be obtained experimentally or by computational toxicology.

To our knowledge, this is the first study providing One Health-oriented threshold concentrations for the integrated protection of human and environmental health. In our experimental conditions, the environmental sector defines the generic HB-TTC, as the eco-TTCs calculated for the 5 Verhaar classes were always lower than the concentrations deriving from the TTC values envisaged for the health assessment of drinking water. This consideration was also valid for the specific HB-TTCs proposed for the non-carcinogenic compounds analyzed, while for carcinogenic compounds the thresholds originate from the concentrations based on the specific parameters used in human health risk assessment.

Finally, we also considered the application of in silico models to predict the (eco)toxicological properties involved in the strategy. These tools are particularly useful for substances with partial or missing profiles. In particular, we proposed some of the models implemented in our in-house VEGAHUB platform that are freely available and can predict exactly the toxicological properties described in the strategy.

## Figures and Tables

**Table 1:** The eco-Threshold of Toxicological Concerns (TTCs) obtained by the described approach. Bold values highlight the lower value of each subset. Values are expressed as ng/L.

Verhaar Class	Empirical Distribution	Normal Fitted Distribution	Logistic Fitted Distribution
1	**5.496**	5.587	5.929
2	**9.455**	37.53	44.45
3	**1.254**	2.534	2.982
4	0.134	0.1433	**0.1175**
5	**4.012**	5.434	5.750

**Table 2:** The hTTC-derived Quality Standards (QS) obtained with the proposed approach. Values are µg/L.

Cramer Class	QS According to the WFD Guideline	QS According to the TTC Approach
1	105	90
2	31.5	27
3	5.25	4.5

**Table 3:** The Human-Biota (HB)-TTCs. Values are ng/L. * indicates that the HB-TTC is derived from logistic fitted distribution.

Chemicals Belonging to Verhaar Class	HB-TTCs
1	5.496
2	9.455
3	1.254
4	0.1175 *
5	4.012

**Table 4:** Comparison of the eco-TTCs in this work with those obtained by Kienzler et al., 2019. Values are ng/L.

Verhaar Class	This Study	Kienzler et al., 2019
1	5.496	45
2	9.455	19
3	1.254	15
4	0.1175	0.2
5	4.012	4

**Table 5:** Examples of models that can be used to predict the eco- and toxicological properties required for the proposed strategy.

Endpoints	Available Models
NOAEL	[36,37,38]
Algae acute toxicity	[39,40,41,42,43,44,45,46]
Algae chronic toxicity	[47,48], Algae Chronic Toxicity VEGA model (see details below)
Daphnia magna acute toxicity	[41,44,46,49,50,51]
Daphnia magna chronic toxicity	[47], Daphnia Chronic Toxicity VEGA model (see details below)
Fish acute toxicity	[34,41,44,46,49,52,53,54,55,56,57,58]
Fish chronic toxicity	Fish Chronic Toxicity VEGA model (see details below)
Consensus models for acute toxicity to aquatic organisms	[59,60]

**Table 6:** Possible data combinations and application factors for Predicted No effect Concentration (PNEC) derivation according to EnviroTox

Data Available and Combination	Assessment Factors
1 trophic acute level	10,000
2 trophic acute levels	5000 on the most sensitive taxon
3 trophic acute levels	1000 on the most sensitive taxon
3 trophic acute levels and 1 chronic data not on the most sensitive acute taxon	1000
3 trophic acute levels and 1 chronic data on the most sensitive acute taxon	100
3 trophic acute levels and 2 chronic data including most sensitive acute taxon	50
3 trophic acute levels and 3 chronic levels	10
More than 10 chronic toxicity data or microcosm/mesocosm studies	1 to 5

**Table 7:** Cramer classes and related TTC values.

Cramer Class	TTC (µg/kgbw d)
1	30
2	9
3	1.5

**Table 8:** Verhaar classes for fish acute toxicity Mechanism of Action (MoA).

Verhaar Class	Mode of Action
1	Narcosis or baseline toxicity
2	Less inert compounds
3	Unspecific reactivity
4	Compounds and groups acting by a specific mechanism
5	Not possible to classify according to these rules (unclassified)

## Data Availability

The list of compound-specific HB-TTCs is available on Zenodo as “Baderna, Diego, & Benfenati, Emilio. (2021). Curated Envirotox database and compound specific Human-Biota TTC from the LIFE+ Vermeer project (Version 1.0) [Data set]. Zenodo. http://doi.org/10.5281/zenodo.4643745”

## Supplementary Materials

The following are available online, SM1 R script for the calculation of the fifth percentile of PNEC distribution; SM2: The curated Envirotox DB (*n* = 3728) with NAME, CAS and SMILES of each chemicals coupled to the Verhaar and Cramer classifications (Excel^®^ file); SM3: Compound-specific HB-TTCs for 410 chemicals (Excel^®^ file); SM4: VEGA models for the prediction of the eco- and toxicological properties required in the proposed strategy and their performance.
