# Supplementary material for: Defining the Human-Biota Thresholds of Toxicological Concern for Organic Chemicals in Freshwater: The Proposed Strategy of the LIFE VERMEER Project Using VEGA Tools
Source: Molecules. 2021 Mar 30;26(7):1928. doi: 10.3390/molecules26071928 (PMC8037015; doi:10.3390/molecules26071928)
Supplement: Supplementary file 1 [file molecules-26-01928-s001.zip › Supplementary/Supplementary.docx]

Article

Defining the human-biota thresholds of toxicological concern for organic chemicals in freshwater: the proposed strategy of the LIFE VERMEER project using VEGA tools.

Diego Baderna ^1,^*, Roberta Faoro ^1^, Gianluca Selvestrel ^1^, Adrien Troise ^2^, Davide Luciani ^1^, Sandrine Andres ^2^ and Emilio Benfenati ^1,^*.

| **Citation:** Lastname, F.; Lastname, F.; Lastname, F. Title. *Molecules* **2021**, *26*, x. https://doi.org/10.3390/xxxxx  Academic Editor: Firstname Lastname  Received: date  Accepted: date  Published: date  **Publisher’s Note:** MDPI stays neutral with regard to jurisdictional claims in published maps and institutional affiliations.  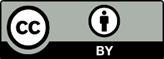  **Copyright:** © 2021 by the authors. Submitted for possible open access publication under the terms and conditions of the Creative Commons Attribution (CC BY) license (http://creativecommons.org/licenses/by/4.0/). |
| --- |

^1^ 1. Laboratory of Environmental Chemistry and Toxicology, Istituto di Ricerche Farmacologiche Mario Negri IRCCS, Italy.

^2^ 2. INERIS Institut National de l'Environnement Industriel et des Risques, France

* Correspondence: D.B. [diego.baderna@marionegri.it](mailto:diego.baderna@marionegri.it) E.B. emilio.benfenati@marionegri.it

**Abstract:** Several tons of chemical agents are released annually into the environment and it is therefore necessary to estimate the risk of inducing adverse effects on human health and ecosystems. This evaluation is based both on the concentration of chemical agent that reaches the target organism and on the eco- and toxicological profile of the substance. Risk assessment is expensive and time-consuming and the information available for so many compounds is still partial. A consolidated approach to overcome this limitation is the use of Threshold of Toxicological Concern (TTC) for the assessment of the potential health impact and, more recently, of eco-TTCs for the ecological aspect. These values are considered as safe concentrations for substances with poor toxicological characterization and low exposure. Only limited attempts have been made to integrate the human and ecological risk assessment procedures in a One Health perspective. In this paper we are proposing a strategy to define the Human-Biota TTCs (HB-TTCs) as concentrations of organic chemicals in freshwater preserving both humans and ecological receptors contemporarily. Two types of thresholds were derived: general HB-TTCs as preliminary screening levels for compounds with no eco- and toxicological information available and compound-specific HB-TTCs for those chemicals with known hazard assessment in terms of PNEC values for freshwater ecosystems and acceptable doses for human health. The proposed strategy is based on freely available public data and tools to characterize and to group chemicals according to their toxicological profiles. Five generic HB-TTCs, based on ecotoxicological profiles as reflected by the Veerhar classes, were defined while compound-specific thresholds were provided for more than 400 organic chemicals with complete eco- and toxicological profiles. To complete the strategy, the use of in silico models is proposed to predict the required toxicological properties and a list of suitable models already available in the VEGAHUB platform is detailed.

List of additional resources

SM1 R script for the calculation of fifth percentile of PNEC distribution

SM2: The curated Envirotox DB (n=3728) with NAME, CAS and SMILES of each chemicals coupled to the Verhaar and Cramer classifications (Excel® file).

SM3: Compound-specific HB-TTCs for 410 chemicals (Excel® file).

SM4: VEGA models for the prediction of the eco and toxicological properties requested in the proposed strategy and their performance.

1. SM1 R script for the calculation of fifth percentile of PNEC distribution with comments (#)

library(fitdistrplus)

*#data upload*

TTCdata<- read.csv(file.path("*your path here*"),sep=";",dec=",",header=T)

*#fifth percentile using empirical distribution*

emp04_perc<- quantile(TTCdata[,2], probs = c(0.05),  na.rm = TRUE)

*#Log transformation*

log10TTC<- log10(TTCdata[,2])

*#fitting on normal distribution*

fln <- fitdist(log10TTC, "norm")

logi05pct_norm<- quantile(fln, probs = c(0.05),  na.rm = TRUE)

*#goodness of fit*

gofstat(fln)

*#95% Confidence intervals*

bln <- bootdist(fln, bootmethod="param", niter=101)

bootEmp05pct_norm<- quantile(bln, probs = c(0.05),  na.rm = TRUE)

CI95_norm<-10^(t(as.vector(bootEmp05pct_norm$quantCI)))

*#plotting data*

cdfcomp(fln)

q1 <- quantile(fln, probs = seq(0,1,length=100))

points(q1$quantCI[1,],q1$probs,type="l")

points(q1$quantCI[2,],q1$probs,type="l")

CIcdfplot(fln, CI.output = "quantile", CI.fill = "pink")

*#fitting on logistic distribution*

flng <- fitdist(log10TTC, "logis" )

*#goodness of fit*

gofstat(flng)

logi05pct_logis<- quantile(flng, probs = c(0.05),  na.rm = TRUE)

*#95% Confidence intervals*

bln_logis <- bootdist(flng, bootmethod="param", niter=101)

bootEmp05pct_logis<- quantile(bln_logis, probs = c(0.05),  na.rm = TRUE)

CI95_logis<-10^(t(as.vector(bootEmp05pct_logis$quantCI)))

*#back transformation of fifth percentile fitted on normal distribution*

o5pct_norm<- 10^(as.numeric(logi05pct_norm[[1]][1]))

*#back transformation of fifth percentile fitted on logistic distribution*

o5pct_logis<- 10^(as.numeric(logi05pct_logis[[1]][1]))

1. SM4: VEGA models for the prediction of the eco and toxicological properties requested in the proposed strategy and their performance.
2. **Models related to human toxicity**

| Name of the model | Type of model | Statistics | Reference |
| --- | --- | --- | --- |
| NOAEL | One-variable model based on 2D descriptor and Monte Carlo method. | Training (n=97)  R^2^ = 0.53  RMSE = 0.61  Calibration (n=16)  R^2^ = 0.73  RMSE = 0.49 | [1]  www.vegahub.eu/vegahub-dwn/qmrf/QMRF_NOAEL_IRFMN.pdf |
| Carcinogenicity oral classification | Classification and regression trees model based on binary classification | Training (n=593)  Accuracy = 0.81  Sensitivity = 0.82  Specificity = 0.79  Test (n= 149)  Accuracy = 0.76 Sensitivity = 0.76 Specificity = 0.76 | [2]  www.vegahub.eu/vegahub-dwn/qmrf/QMRF_SFO_CLASS.pdf |
| Carcinogenicity oral Slope Factor | Multi-layer perceptron – artificial neural networks (MLP-ANNs) with oral SF values from RAIS | Training (n= 283)  R2 = 0.661  RMSE = 0.956  Test (n=32)  R2 = 0.573  RMSE = 1.28 | [2]  www.vegahub.eu/vegahub-dwn/qmrf/QMRF_SFO_REG.pdf |

1. **Models for algal toxicity**

| Name of the model | Type of model | Statistics | Additional information |
| --- | --- | --- | --- |
| Algae acute toxicity | Tree Ensemble Random Forest with 72h ErC50 data | Training (n=252)  R^2^= 0.82  RMSE = 0.85  Test (n=63)  R^2^= 0.79  RMSE = 0.87 | www.vegahub.eu/vegahub-dwn/qmrf/QMRF_ALGAE_EC50_IRFMN.pdf |
| Algae chronic toxicity | Tree Ensemble Random Forest with 72h NOEC data based on growth rate | Training (n=652)  R^2^ = 0.97  RMSE = 0.92  Test (n=163)  R^2^ = 0.63  RMSE = 0.89 | www.vegahub.eu/vegahub-dwn/qmrf/QMRF_ALGAE_NOEC_IRFMN.pdf |

1. Models for daphnia toxicity

| Name of the model | Type of model | Statistics | Additional information |
| --- | --- | --- | --- |
| *Daphnia magna* acute toxicity (DEMETRA) | Hybrid model based on multiple linear regressions with 48h EC50 data according to OECD 202 guideline | Training (n=220)  R2 = 0.75  Test (n=43)  R2 = 0.68  RMSE = 1.49 | [3]  www.vegahub.eu/vegahub-dwn/qmrf/QMRF_Daphnia_EC50_IRFMN.pdf |
| *Daphnia* Acute (EC50) toxicity (IRFMN) | Tree Ensemble Random Forest with 48h EC50 data according to OECD 202 guideline | Training (n=312)  R2 = 0.68  RMSE = 0.62  Test (n=133)  R2 = 0.7  RMSE = 061 | www.vegahub.eu/vegahub-dwn/qmrf/QMRF_Daphnia_EC50_IRFMN.pdf |
| *Daphnia magna* chronic toxicity | Tree Ensemble Random Forest.  with data according to the OECD TG 211 requirements | Training (n=215)  R^2^ = 0.64  RMSE =0.71  Test (n=92)  R^2^ = 0.5  RMSE = 0.81 | www.vegahub.eu/vegahub-dwn/qmrf/QMRF_Daphnia_NOEC_IRFMN.pdf |

1. **Models for fish toxicity**

| Name of the model | Type of model | Statistics | Additional information |
| --- | --- | --- | --- |
| Fish Acute (LC50) Toxicity model (NIC) | Counter Propagation Artificial Neural  Network (CP ANN) with 96h LC50 values for four species *(Oncorhynchus mykiss, Oryzias latipes, Pimephales promelas and Poecilia reticulata).* | Training (n=564)  R^2^ = 0.89  RMSE = 0.42  Test (n=382)  R^2^ = 0.49  RMSE = 0.90 | www.vegahub.eu/vegahub-dwn/qmrf/QMRF_FISH_LC50_NIC.pdf |
| Fish Acute (LC50) Toxicity model (IRFMN) | Tree Ensemble Random Forest with 96h LC50 values from studies with *Oryzias latipes* following OECD 203. | Training (n=264)  R^2^ = 0.72  RMSE = 0.75  Test (n=67)  R^2^ = 0.51  RMSE = 0.96 | www.vegahub.eu/vegahub-dwn/qmrf/QMRF_FISH_LC50_IRFMN.pdf |
| Fathead Minnow LC50 model | k nearest neighbor (kNN) model with 96h LC50 data with *Pimephales promelas.* | Statistics obtained by leave-one-out cross-validation: n=602  R^2^ = 0.69  RMSE = 0.78 | www.vegahub.eu/vegahub-dwn/qmrf/QMRF_FATHEAD_LC50_KNN.pdf |
| Fish chronic toxicity | Tree Ensemble Random Forest with data according to OECD TGs 210, 212 and 215 requirements. | Training (n=652)  R^2^ = 0.97  RMSE = 0.92  Test (n=163)  R^2^ = 0.63  RMSE = 0.89 | www.vegahub.eu/vegahub-dwn/qmrf/QMRF_FISH_NOEC_IRFMN.pdf |

1. References

[1] Toropov, A.A., Toropova, A.P., Pizzo, F., Lombardo, A., Gadaleta, D., Benfenati, E., 2015. CORAL: model for no observed adverse effect level (NOAEL). Mol Divers 19, 563–575. <https://doi.org/10.1007/s11030-015-9587-1>

[2] Toma, C., Manganaro, A., Raitano, G., Marzo, M., Gadaleta, D., Baderna, D., Roncaglioni, A., Kramer, N., Benfenati, E., 2021. QSAR Models for Human Carcinogenicity: An Assessment Based on Oral and Inhalation Slope Factors. Molecules 26, 127. <https://doi.org/10.3390/molecules26010127>

[3] Amaury, N., Benfenati, E., Boriani, E., Casalegno, M., Chana, A., Chaudhry, Q., Chrétien, J.R., Cotterill, J., Lemke, F., Piclin, N., Pintore, M., Porcelli, C., Price, N., Roncaglioni, A., Toropov, A., 2007. Chapter 7 - Results of DEMETRA models, in: Benfenati, EMILIO (Ed.), Quantitative Structure-Activity Relationships (QSAR) for Pesticide Regulatory Purposes. Elsevier, Amsterdam, pp. 201–281. <https://doi.org/10.1016/B978-044452710-3/50009-4>
